# Altered Expression of Energy Metabolism-Related Genes in Placental Trophoblast Cells in Preeclampsia

**DOI:** 10.33549/physiolres.935812

**Published:** 2026-08-01

**Authors:** Jing WANG, Xin WANG, Ling-Chao WANG, Mei-Li HU, Wei LIU, Jie CUI, Hong-Jie LI, Xian ZHENG

**Affiliations:** 1 Department of Obstetrics and Gynecology, Affiliated Hospital of Hebei University, Baoding, Hebei, China; 2 Baoding Maternity and Child Health Care Hospital, Baoding, Hebei, China; 3 Medical Company of PLA, Baoding, Hebei, China

**Keywords:** Glycolysis, PGC-1α, Placenta, Preeclampsia, SIRT-1

## Abstract

Preeclampsia (PE) is a pregnancy-specific hypertensive disorder characterized by new-onset hypertension after 20 weeks of gestation, with or without proteinuria, frequently accompanied by systemic arteriolar spasm and dysfunction or injury of multiple organs, including the liver, kidneys, and placenta. This study examined changes in glycolysis- and mitochondria-related gene expression in placental trophoblast cells in PE, aiming to elucidate the role of disrupted energy metabolism in its pathogenesis. A total of 86 pregnant women with singleton pregnancies who received routine prenatal care at the Affiliated Hospital of Hebei University between December 2023 and October 2024 and delivered *via* cesarean section were enrolled. Among them, 30 patients diagnosed with PE were included in the PE group, and 56 normotensive pregnant individuals were assigned to the control group. General clinical data were collected. The mRNA and protein expression levels of HK2, PKM2, silent information regulator two 1 (SIRT1), and peroxisome proliferator-activated receptor gamma coactivator 1-alpha (PGC-1α) in placental tissue were measured using real-time quantitative polymerase chain reaction and western blot (WB) analysis. There were no statistically significant differences in maternal age at delivery, body mass index, triglycerides, total cholesterol, or high-density lipoprotein cholesterol between groups (*p* > 0.5). Significant differences were observed in low-density lipoprotein cholesterol, neonatal birth weight, and 1-minute and 5-minute Apgar scores (*p* < 0.05). Expression levels of HK2, PKM2, and PGC-1α were significantly elevated in placental tissue from the PE group compared to the control group (*p* < 0.01), while SIRT1 expression was significantly reduced (*p* < 0.05). Placental tissue from patients with PE demonstrated upregulation of *HK2*, *PKM2*, and *PGC-1α*, and downregulation of *SIRT1*. Dysregulated expression of genes and proteins involved in energy metabolism may contribute to the pathogenesis of PE through impaired trophoblast function.

## Introduction

Preeclampsia (PE) is a common pregnancy complication, with an incidence of approximately 3 %–8 % [1]. It is primarily characterized by hypertension, frequently accompanied by proteinuria and maternal organ dysfunction, and is associated with substantial risks to pregnant patients and their fetuses, often leading to iatrogenic preterm delivery and more severe adverse pregnancy outcomes. With advances in research on PE pathogenesis, placental dysfunction has been recognized as a key contributing factor. The “two-stage model” proposed by Redman et al. in 2009 indicated that trophoblast cell dysfunction develops as a consequence of ischemia and hypoxia in early pregnancy, resulting in inadequate remodeling of uterine spiral arteries and subsequent placental dysfunction [2]. The subsequent excessive release of placenta-derived factors contributes to an amplified maternal inflammatory response and vascular endothelial injury, leading to a spectrum of clinical manifestations. In 2014, Redman further refined this framework by proposing the “six-stage model,” emphasizing PE pathogenesis from the perspective of pathophysiological changes in placental trophoblast cells [3]. Jaremek et al. reported reduced proliferative and invasive capacities of placental trophoblast cells in PE, which were consistent with these findings [4].

Placental trophoblast cells predominantly use high-rate glycolysis to fulfill the energy requirements necessary for rapid proliferation and invasion under aerobic conditions. Therefore, this study examined the energy metabolism profile of placental trophoblast cells in PE, with the objective of identifying potential targets for therapeutic intervention.

## Materials and Methods

### General data

Thirty pregnant women with PE who delivered at the Affiliated Hospital of Hebei University between December 2023, and October 2024 were enrolled as the study group (PE group). The mean maternal age was (27.13 ± 0.71) years, mean gestational age was (36.00 ± 0.31) weeks, and mean neonatal birth weight was (2725 ± 140.5) g. The 1-minute and 5-minute neonatal Apgar scores were (9.03 ± 0.16) and (9.47 ± 0.09), respectively. Laboratory measurements showed low-density lipoprotein cholesterol (LDL-C) levels of (3.23 ± 0.18) mmol/L, triglyceride (TG) levels of (3.37 ± 0.25) mmol/L, total cholesterol (TC) levels of (6.11 ± 0.23) mmol/L, and high-density lipoprotein cholesterol (HDL-C) levels of (1.84 ± 0.07) mmol/L.

During the same period, 56 pregnant women who underwent cesarean delivery without pregnancy-related or other complications were selected as the control group (primary group). In this group, the mean maternal age was (27.38 ± 0.46) years, mean gestational age was (39.20 ± 0.09) weeks, and mean neonatal birth weight was (3505 ± 43.58) g. The 1-minute and 5-minute neonatal Apgar scores were (9.79 ± 0.07) and (9.98 ± 0.02), respectively. LDL-C levels were (2.72 ± 0.13) mmol/L, TG at (3.00 ± 0.14) mmol/L, TC were (5.82 ± 0.15) mmol/L, and HDL-C levels were (1.98 ± 0.06) mmol/L. Statistical comparisons between groups is presented in Table 1.

### Inclusion and exclusion criteria

Inclusion criteria: (1) Pregnant women with PE who had no prior history of cardiac, hepatic, renal, or endocrine disorders; no pregnancy-related complications or other comorbid conditions; and no premature rupture of membranes or infections during the current pregnancy. Diagnostic criteria for PE were applied according to the 9th edition of *Obstetrics and Gynecology* published by People’s Medical Publishing House [5]. (2) Ethical approval for the study was granted by the Ethics Committee of the Affiliated Hospital of Hebei University. Participation was voluntary, and written informed consent was obtained from all pregnant women and their family members.

Exclusion Criteria: (1) Presence of multiple pregnancy, conception via assisted reproductive technology, fetal or placental structural abnormalities, or fetal chromosomal abnormalities; (2) Pregnancy complicated by medical or surgical conditions, such as cardiovascular disease, renal disease, or pancreatitis; (3) History of congenital disorders or psychiatric illness.

### Specimen collection

Within 30 minutes following placental delivery during cesarean section, placental tissue measuring approximately 1 cm × 1 cm × 1 cm was excised under sterile conditions from the maternal surface near the umbilical cord insertion site, with care taken to avoid large blood vessels and areas of calcification. Residual blood was removed by repeated rinsing with precooled normal saline. The tissue specimens were then transferred into cryovials and preserved in liquid nitrogen. After rapid freezing, the samples were stored at −80 °C for subsequent analysis.

### RT-PCR detection of related gene expression levels in placental tissue

(1) Total RNA was extracted from placental tissue using the TRIzol method in accordance with the manufacturer’s instructions. Complementary DNA (cDNA) was synthesized via reverse transcription, and polymerase chain reaction (PCR) amplification was subsequently performed using cDNA as the template, following the protocol provided with the commercial kit. The reaction system included 1 μL cDNA template, 0.8 μL each of forward and reverse primers, 10 μL SuperStar Universal SYBR Master Mix, and 7.4 μL of sterile water. The PCR conditions were as follows: initial denaturation at 95 °C for 30 seconds; denaturation at 95 °C for 10 seconds; annealing/extension at 60 °C for 20 seconds; melting at 95 °C for 15 seconds; and 60 °C for 1 minute, for a total of 45 cycles. Each sample was analyzed in triplicate.

(2) PCR primer sequences

*β-actin* was used as the internal reference gene. The primer sequences were as follows:

Hexokinase 2 (HK2) F 5’-GAGTTTGACCTGGA TGTGGTTGC-3’
R 5’-CCTCCATGTAGCAGGCATTGCT-3’
SIRT-1 F 5’-TAGACACGCTGGAACAGGTTGC-3’
R 5’-CTCCTCGTACAGCTTCACAGTC-3’
Pyruvate kinase M2 (PKM2) F 5’-ATGGCTGACAC ATTCCTGGAGC-3’
R 5’-CCTTCAACGTCTCCACTGATCG-3’
PGC-1α F 5’-CCAAAGGATGCGCTCTCGTTCA-3’
R 5’-CGGTGTCTGTAGTGGCTTGACT-3’
β-actin F 5’-TGACGTGGACATCCGCAAAG-3’
R 5’-CTGGAAGGTGGACAGCGAGG-3’

(3) PCR result analysis

After completion of the amplification, the Ct values for each reaction well were recorded. Relative gene expression levels were calculated using the 2^-ΔΔCt method. Results were expressed as mean ± standard deviation (*χ̄* ± s).

### Western blot

Placental tissue was washed 2–3 times with phosphate-buffered saline, cut into small fragments, and transferred into homogenization tubes. Two 4-mm homogenization beads were added to each sample, followed by the addition of lysis buffer at a volume 10 times that of the tissue. Homogenization was then performed. The resulting homogenates were centrifuged at 12,000 rpm at 4 °C for 10 minutes, and the supernatant was collected. Protein concentration was quantified using a bicinchoninic acid protein assay kit, according to the instructions provided by the manufacturer. Equal amounts of total protein were separated by SDS-PAGE and transferred onto 0.45-μm polyvinylidene difluoride membranes. Membranes were blocked for 30 minutes and subsequently incubated overnight at 4 °C with primary antibodies against HK2 (1:1000), PKM2 (1:1000), silent information regulator two 1 (SIRT1) (1:1000), peroxisome proliferator-activated receptor gamma coactivator 1-alpha (PGC-1α) (1:1000), and the internal control β-actin (1:10000) on a shaker. After three washes with Tris-buffered saline containing Tween-20, membranes were incubated with a 1:5000-diluted secondary antibody at room temperature for 30 minutes. Protein bands were visualized using a chemiluminescence imaging system, and band intensities were analyzed using AIWBwell™ analysis software. Each experiment was performed in triplicate.

### Statistical methods

Data analysis and figure generation were conducted using SPSS 23.0 software and GraphPad Prism 8. Quantitative data conforming to a normal distribution were expressed as mean ± standard deviation. Comparisons between two groups were performed using the independent samples *t*-test. A value of *p* < 0.05 was considered statistically significant.

## Results

### Expression of SIRT1 in PE placental tissue

**Fig. 1:**
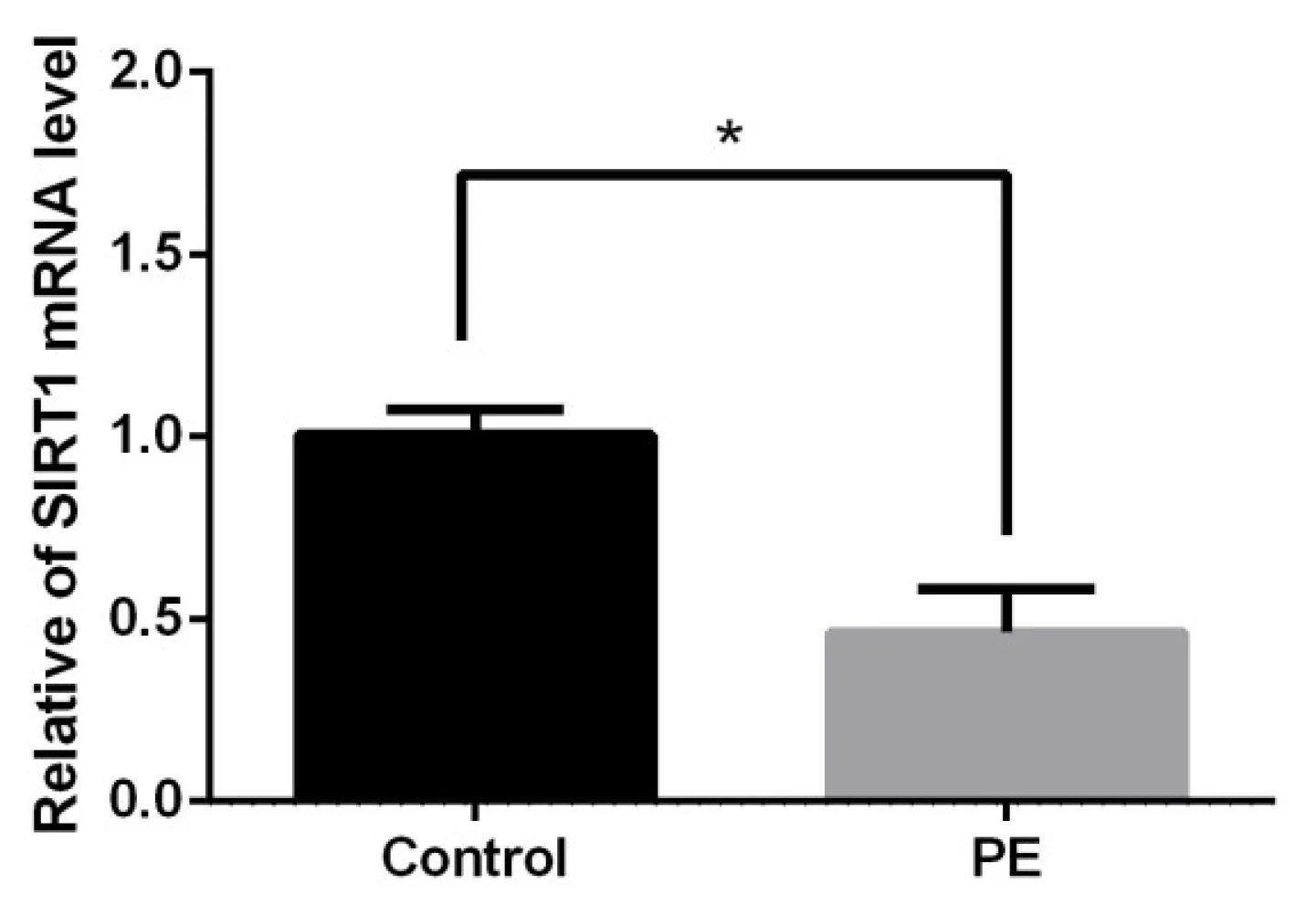
*SIRT1* mRNA expression in placental trophoblast cells from patients with and without PE (PE and Control groups). Relative mRNA levels of *SIRT1* were measured by real-time quantitative PCR. Statistical analysis was performed using Student’s *t*-test (*p* = 0.016). The assay was conducted in triplicate.

A significantly lower level of mRNA expression was observed in placental tissue from the PE group compared with the control group (*p* < 0.05). Refer to Figure 1.

### Expression of PGC-1α in PE placental tissue

**Fig. 2:**
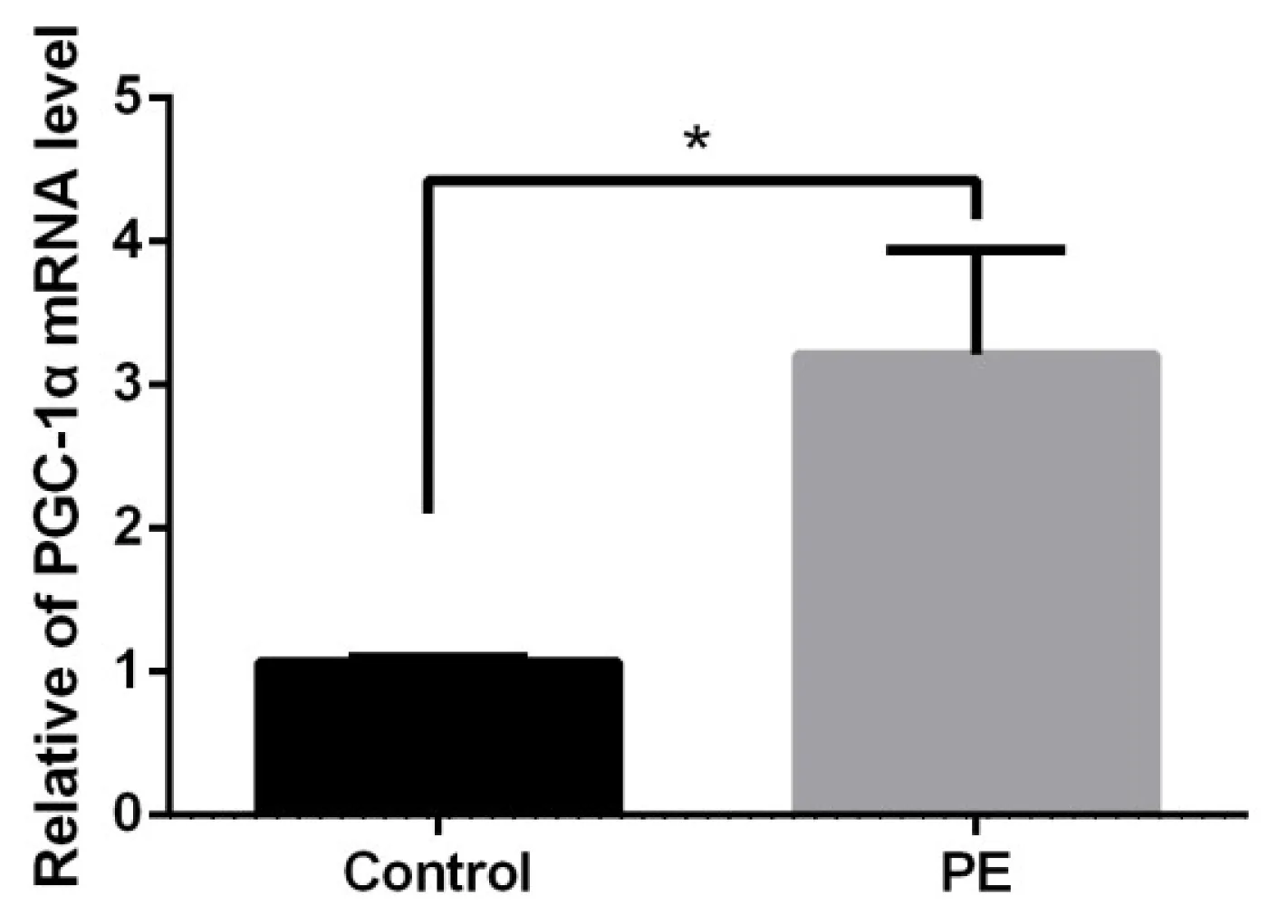
*PGC-1α* mRNA expression in placental trophoblast cells from patients with and without PE (PE and Control groups). Relative mRNA levels of *PGC-1α* were measured by real-time quantitative PCR. Statistical analysis was conducted using Student’s *t*-test (*p* = 0.042). The assay was conducted in triplicate.

A significantly higher level of *PGC-1α* mRNA expression was observed in placental tissue from the PE group compared with the control group (*p* < 0.05). Refer to Figure 2.

### Expression of HK2 and PKM2 in PE placental tissue

**Fig. 3:**
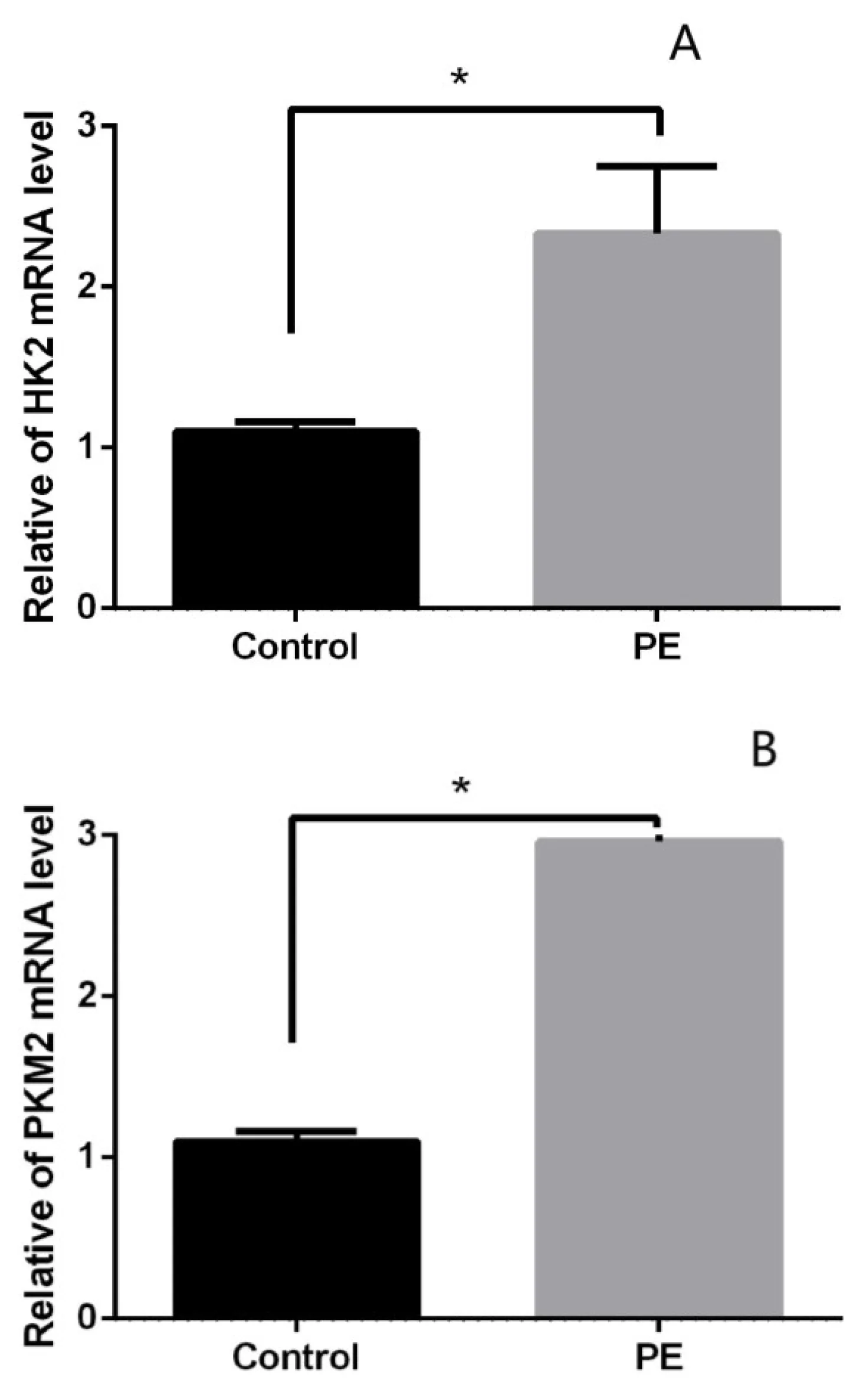
(**A–B**) *HK2* and *PKM2* mRNA expression in placental trophoblast cells from patients with and without PE (PE and Control groups). Relative mRNA levels of *HK2* and *PKM2* were measured by real-time quantitative PCR. Statistical analysis was conducted using Student’s *t*-test (A: *p* = 0.043; B: *p* = 0.041). The assay was conducted in triplicate.

Significantly higher mRNA expression levels of *HK2* and *PKM2* were observed in placental tissue from the PE group compared with the control group (*p* < 0.05). Refer to Figure 3.

### Comparison of SIRT-1, PGC-1α, HK2, and PKM2 protein expression levels in PE placental tissue

**Fig. 4:**
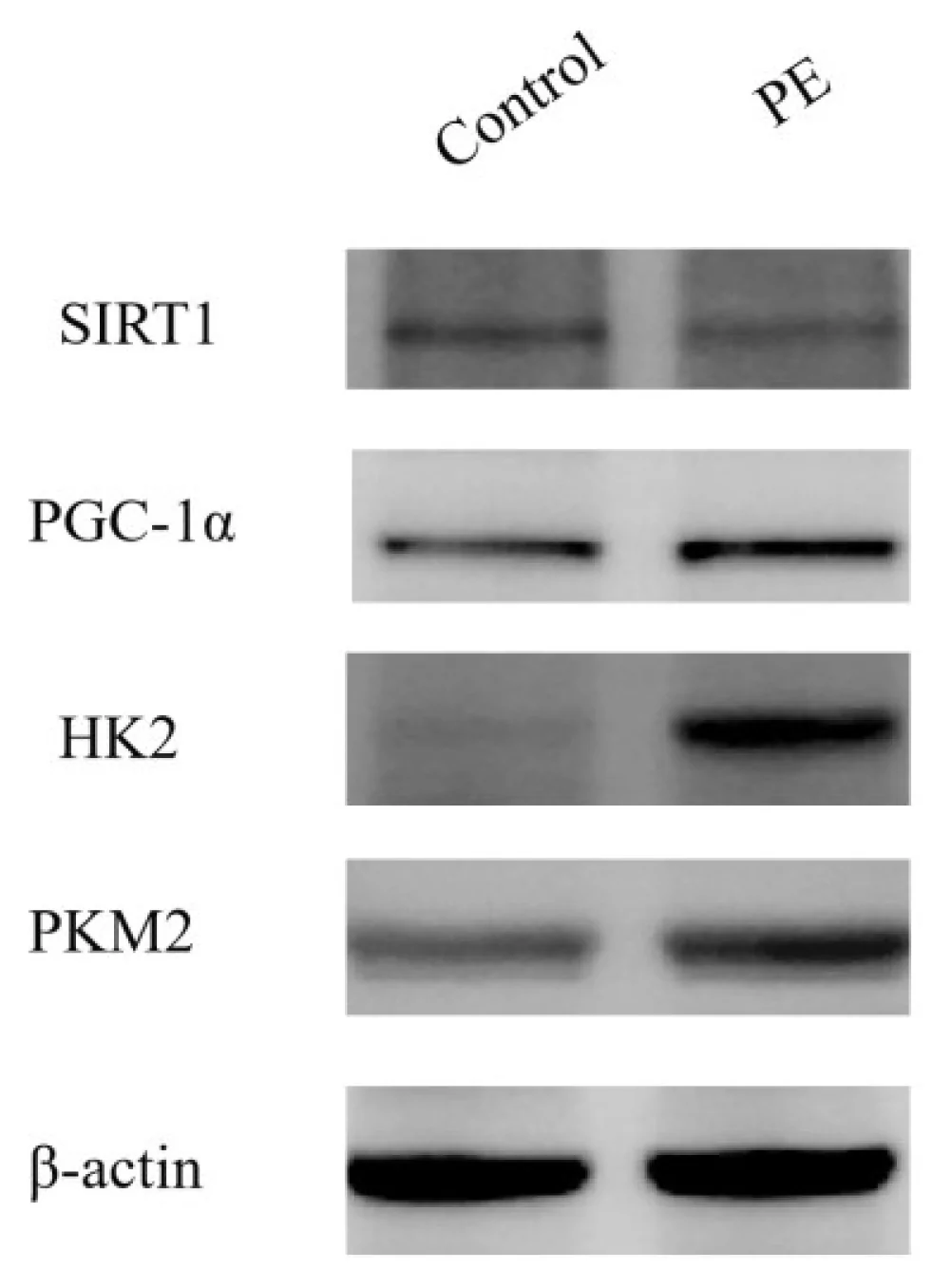
WB analysis of SIRT1, PGC-1α, HK2, and PKM2 protein expression in placental tissue from the PE and Control groups. SIRT1 protein expression was reduced in the PE group, while expression levels of PGC-1α, HK2, and PKM2 were elevated compared with the Control group. Statistical analysis was performed using Student’s *t*-test (*p* < 0.05). The assay was conducted in triplicate.

To assess the protein expression levels of PGC-1α, SIRT1, HK2, and PKM2 in human placental trophoblast cells, placental tissue samples from six patients with PE and six normotensive pregnant individuals were analyzed. WB was conducted to detect protein expression levels of PGC-1α, SIRT1, HK2, and PKM2 (Fig. 4). Reduced expression of SIRT1 protein was observed in placental tissue from the PE group, while protein levels of PGC-1α, HK2, and PKM2 were increased in the same group.

## Discussion

PE is a progressive, pregnancy-related disorder defined by new-onset hypertension occurring after 20 weeks of gestation, with or without proteinuria, and is associated with damage or dysfunction in multiple organ systems, including the liver, kidneys, and placenta [6]. In severe cases, the condition may become life-threatening for both the pregnant patient and the fetus. Globally, PE accounts for an estimated 76,000 maternal deaths and 500,000 infant deaths annually, with an incidence ranging from 2 % to 8 % [7,8]. Currently, the only effective intervention involves timely termination of pregnancy and delivery of both the fetus and placenta [9,10]. Consequently, substantial evidence supports the classification of PE as a placenta-originated disorder, with changes in energy metabolism within placental trophoblast cells implicated in its pathogenesis [11,12].

Previous studies have indicated that abnormal differentiation of extra villous trophoblast cells during placental development contributes to the pathogenesis of PE. These cells use substantial amounts of glucose and fatty acids, thereby influencing placental maturation and function [13]. Mitochondrial ATP deficiency within placental trophoblast cells may result in placental dysfunction, which subsequently impairs maternal organ function and contributes to the clinical manifestations of PE. Therefore, placental dysfunction is considered a central factor in the development of PE, and investigation of energy metabolism changes in placental trophoblast cells remains critical to understanding its underlying mechanisms [14].

Normal trophoblast cells are capable of flexibly using glycolysis to support their invasive and proliferative capacities. Xing et al. reported abnormally elevated GLUT3 expression in placental tissue from patients with PE, facilitating increased placental glucose transport [15–17]. Similarly, Phoswa et al. identified altered glucose transport in placental tissue from patients with PE [18]. In PE, the metabolic profile of trophoblast cells becomes dysregulated, as evidenced by significantly enhanced glycolytic activity. In this study, glycolysis-related genes, including *HK2* and *PKM2*, were upregulated in placental trophoblast cells from the PE group. Along with this increased glycolytic activity, mitochondrial dysfunction, excessive production of reactive oxygen species, and upregulation of the NLRP3 inflammasome were observed in the PE group, findings that are closely associated with the heightened inflammatory responses characteristic of PE [19]. As the migration and invasion of placental trophoblast cells are highly energy-dependent, metabolic disturbances can impair intracellular signaling pathways, thereby contributing to the initiation and progression of PE.

In this study, PCR and WB analyses demonstrated reduced SIRT1 expression in placental tissue from patients with PE. These findings were consistent with those reported by Pei et al., in which pregnant mice with SIRT1 knockdown exhibited PE-like manifestations [20]. Similarly, Zhou et al. reported significantly decreased *SIRT1* mRNA expression in placental tissue from patients with PE [21]. Viana-Mattioli et al. further demonstrated reduced plasma SIRT1 levels in patients with PE, as well as decreased SIRT1 expression in HUVECs incubated with plasma from patients with PE [22]. SIRT1 are closely associated with trophoblast cell invasion, proliferation, differentiation, and apoptosis. Studies conducted by Cao and Li et al. using HTR8/SVneo cell models indicated that SIRT1 knockout significantly reduced proliferative, invasive, and migratory capacities of placental trophoblast cells, while apoptosis was increased [23,24]. Therefore, reduced SIRT1 expression in placental trophoblast cells may contribute to the development and progression of PE.

*PGC-1α* is recognized as a key regulator of mitochondrial metabolism. It participates in and regulates various cellular biological processes, including energy metabolism, primarily through the control of mitochondrial biogenesis and function. By modulating multiple downstream genes and signaling pathways, PGC-1α promotes mitochondrial biogenesis and contributes to the maintenance of mitochondrial function. In recent years, the SIRT1/PGC-1α axis has become a focus of investigation due to its key role in angiogenesis; however, its involvement in PE has been infrequently reported. In contrast to the reduced expression of SIRT1, both mRNA and protein expression levels of PGC-1α were elevated in placental trophoblast cells from patients with PE. This elevation may reflect a compensatory regulatory response. During mid-to-late gestation, increased PGC-1α expression in placental trophoblast cells may enhance mitochondrial function and activity to meet the elevated energy demands associated with fetal growth and development, thereby partially compensating for mitochondrial metabolic dysfunction in the context of PE. Finck reported that upregulation of PGC-1α improves mitochondrial function, restores cellular energy metabolism homeostasis, and may exert therapeutic effects in disease states [25]. Based on these findings, it is plausible that decreased SIRT1 expression in combination with increased PGC-1α expression contributes to the development and progression of PE.

## Conclusion

Trophoblast cells serve as the primary functional units of the placenta and are essential for embryo implantation, placental development, and the exchange of substances between the maternal and fetal circulations. Findings from this study indicate that abnormalities in energy metabolism are present in placental trophoblast cells in PE. Mitochondrial dysfunction, along with altered glycolytic activity in these cells, may constitute an early event in the pathogenesis of PE.

## Figures and Tables

**Table 1:** Comparison of patients’ characteristics in both groups (mean ± standard deviation)

Parameters	Control	PE	P value
*Number of cases*	56	30	
*Age (year)*	27.38 ± 0.46	27.13 ± 0.71	0.77
*BMI (Kg/m2)*	34.03 ± 0.46	35.05 ± 0.80	0.24
*Gestation (week)*	39.20 ± 0.09	36.00 ± 0.31	<0.0001***
*Neonatal weight (g)*	3505 ± 43.58	2725 ± 140.5	<0.0001***
*Apgar scor (1min)*	9.79 ± 0.07	9.03 ± 0.16	<0.0001***
*Apgar scor (5min)*	9.98 ± 0.02	9.47 ± 0.09	<0.0001***
*TG (Mmol/L)*	3.00 ± 0.14	3.37 ± 0.25	0.19
*TC(Mmol/L)*	5.82 ± 0.15	6.11 ± 0.23	0.29
*LDL-C (Mmol/L)*	2.72 ± 0.13	3.23 ± 0.18	0.02*
*HDL-C (Mmol/L)*	1.98 ± 0.06	1.84 ± 0.07	0.16

## Abbreviations

PE: preeclampsia
LDL-C: low-density lipoprotein cholesterol
TG: triglyceride
TC: total cholesterol
HDL-C: high-density lipoprotein cholesterol
BMI: body mass index
PGC-1α: peroxisome proliferator activated receptor γ coactivator 1 α
ROS: Reactive oxygen species
CCCP: Carbonyl cyanidem-chlorophenyl hydrazine
BA: Bromopyruvic acid
qRT-PCR: Quantitative Real-time polymerase chain reaction
